# The bacterial virulence factor CagA induces microbial dysbiosis that contributes to excessive epithelial cell proliferation in the *Drosophila* gut

**DOI:** 10.1371/journal.ppat.1006631

**Published:** 2017-10-19

**Authors:** Tiffani Alvey Jones, Diane Z. Hernandez, Zoë C. Wong, Anica M. Wandler, Karen Guillemin

**Affiliations:** 1 Institute of Molecular Biology, University of Oregon, Eugene, OR, United States of America; 2 Humans and the Microbiome Program, Canadian Institute for Advanced Research, Toronto, Ontario, Canada; Stanford University School of Medicine, UNITED STATES

## Abstract

Gut microbiota facilitate many aspects of human health and development, but dysbiotic microbiota can promote hyperplasia and inflammation and contribute to human diseases such as cancer. Human patients infected with the gastric cancer-causing bacterium *Helicobacter pylori* have altered microbiota; however, whether dysbiosis contributes to disease in this case is unknown. Many *H*. *pylori* human disease phenotypes are associated with a potent virulence protein, CagA, which is translocated into host epithelial cells where it alters cell polarity and manipulates host-signaling pathways to promote disease. We hypothesized that CagA alone could contribute to *H*. *pylori* pathogenesis by inducing microbial dysbiosis that promotes disease. Here we use a transgenic *Drosophila* model of CagA expression to genetically disentangle the effects of the virulence protein CagA from that of *H*. *pylori* infection. We found that expression of CagA within *Drosophila* intestinal stem cells promotes excess cell proliferation and is sufficient to alter host microbiota. Rearing CagA transgenic flies germ-free revealed that the dysbiotic microbiota contributes to cell proliferation phenotypes and also elicits expression of innate immune components, Diptericin and Duox. Further investigations revealed interspecies interactions are required for this dysbiotic CagA-dependent microbiota to promote proliferation in CagA transgenic and healthy control *Drosophila*. Our model establishes that CagA can alter gut microbiota and exacerbate cell proliferation and immune phenotypes previously attributed to *H*. *pylori* infection. This work provides valuable new insights into the mechanisms by which interactions between a specific virulence factor and the resident microbiota can contribute to the development and progression of disease.

## Author summary

Microbial communities in the gut, termed microbiota are important for human health, and when altered can sometimes promote disease. Infections, such as with the cancer-causing bacterium *Helicobacter pylori*, can cause altered gut microbiota, but why these alterations occur and whether the altered communities contribute to disease remain unknown. Here, we use *Drosophila* expressing the *H*. *pylori* disease-causing protein CagA, to model this virulence factor’s effect on host pathology and microbiota. We found that expression of CagA in the *Drosophila* gut causes excessive cell proliferation and immune activation, hallmarks of *H*. *pylori* infection. Notably, these traits did not occur when flies were reared in the absence of microbes. Further examination reveals that CagA-expressing flies have an altered gut microbial community that is sufficient to promote cell proliferation even in normal flies. This proliferative activity required the presence of two interacting bacteria, illustrating a new model for disease-promoting microbiota. This work demonstrates how a bacterial protein can cause disease indirectly through altering the microbial ecology of the host, and it suggests future treatments for infections that rely on manipulating the microbiota to mitigate disease pathology.

## Introduction

Gut microbiota, a complex consortium of bacteria, archaea, viruses and eukaryotes found in the gut, play critical roles in human health. The microbiota is known to facilitate nutrient acquisition, confer resistance to pathogens, and contribute to developmental processes [1]. Pathologic alterations in microbial community composition, termed dysbiosis, result in community dysfunction that is linked to human diseases including inflammatory bowel disease, obesity, pathogen infection and colon cancer [2–5]. Indeed, dysbiotic gut microbiota have the ability to alter immune surveillance mechanisms, and promote proliferation and chronic inflammation within the gastrointestinal tract, processes that play key roles in carcinogenesis [6].

Our current understanding of the mechanisms of microbiota maintenance or induced dysbiosis is limited, but host, environmental, and microbial factors can all influence microbiome structure and interactions [5]. For example, host factors such as chronic inflammation [4] and decreased gut motility [7] modulate microbial community composition to promote disease. Environmental perturbations such as diet and antibiotic usage can allow overgrowth of single pathobionts, such as *C*. *difficle* [8]. Conversely, infection with single pathogens can result in altered microbiota composition [3]. For example, S*almonella enterica serovar Typhimurium* promotes and thrives on enteric inflammation, creating an environment that also favors other inflammation-adapted Proteobacteria. Alteration in community composition can also enhance virulence of pathogenic microbes. A notable example comes from mice where microbiota transplants from a susceptible population to a previously unsusceptible population conferred susceptibility to the pathogen *C*. *rodentium* [9].

*Helicobacter pylori* is a bacterial resident of the human stomach that in half the world’s population promotes gastritis and an increased risk for gastric cancer development. Infection with *H*. *pylori* is associated with shifts in the gastric and colonic microbiota [10], but the extent to which *H*. *pylori*-induced dysbiosis contributes to disease is not known. The bacterial-intrinsic mechanisms by which *H*. *pylori* exhibits its oncogenic potential are thought to be largely through expression of a potent virulence protein, cytotoxin-associated gene A (CagA), which is injected into the cytoplasm of host gastric epithelial cells [11]. Upon gaining entry to host cells, CagA modulates multiple host pathways such as the Ras/ERK MAPK pathway, resulting in disruption of receptor tyrosine kinase signaling and promotion of cell proliferation [12]. Additionally, CagA activates inflammatory processes through the immune effector NF-κB, which promotes expression of pro-inflammatory cytokines and alters the host response to infection [13].

Profiling of gastric and colonic microbiota in *H*. *pylori*-infected humans and rodent models demonstrates that this bacterium has a profound effect on resident microbial communities [10,14]. In a mouse model of *H*. *pylori* infection, composition of microbiota prior to *H*. *pylori* colonization impacted disease severity and progression, suggesting gastric microbiota modulate *H*. *pylori* virulence and contribute to gastric disease [15]. Similarly, *H*. *pylori* mono-associated INS-GAS mice showed significantly delayed tumor development and less severe gastritis than when reared with conventional microbiota and *H*. *pylori* [16], again suggesting gastric microbiota play a role in disease pathogenesis. Although the direct role of *H*. *pylori* mediated gastric disease has been well studied, mechanisms by which the *H*. *pylori*-associated gastric microbiota promote disease remain unexplored.

Based on these findings, we hypothesized that the *H*. *pylori* virulence protein CagA contributes to pathological proliferation and promotes carcinogenesis via modulation of gastric microbiota, which we tested in a transgenic *Drosophila* model of CagA expression. Here we took advantage of the relative simplicity of the *Drosophila* midgut epithelia, microbial community, and genetics to transgenically express CagA within the adult midgut epithelium to investigate the potential effect of CagA expression on resident microbiota. The fruit fly midgut shares many similarities with the mammalian gastrointestinal tract in its tissue organization and programs of homeostasis [17]. Both tissues are continually renewing epithelia with stem cell populations that give rise to transit amplifying cells (called enteroblasts in *Drosophila*) that then differentiate into absorptive or secretory cell populations. Also like the mammalian digestive tract, the fruit fly gut contains specialized compartments, including an acidified middle midgut that contains specialized H^+^/K^+^-ATPase-expressing, acid-secreting Copper cells that function similarly to the parietal cells of the human stomach. In addition to similarities in tissue architecture, many of the same molecular pathways, including Wnt and Notch signaling, regulate these programs of epithelial renewal in flies and mammals. Using the *Drosophila* model we recapitulated known host cellular phenotypes of CagA expression and explored, for the first time, a role for resident microbiota as a contributor to pathological proliferation mediated by CagA.

## Results

### Transgenic expression of CagA in the *Drosophila* midgut recapitulates known disease phenotypes associated with CagA

We previously developed the *Drosophila* model of transgenic expression of CagA to elucidate the biochemical and cellular interactions that facilitate pathogenicity of CagA in model epithelial tissues. Specifically our analysis of transgenic expression of CagA in *Drosophila* photoreceptors revealed CagA’s function as a prokaryotic mimic of the Grb2-associated binder (Gab) adaptor protein that activates SHP-2, a component of receptor tyrosine kinase pathways [18]. Additionally, transgenic expression of CagA in the *Drosophila* wing and eye imaginal discs revealed that CagA triggers JNK pathway activation and acts to enhance tumor growth and metastasis generated by activated RAS [19]. Here we use transgenic expression of CagA in the adult midgut epithelium to investigate the affects of CagA on the gut epithelium and host-associated microbiota.

**Fig 1:**
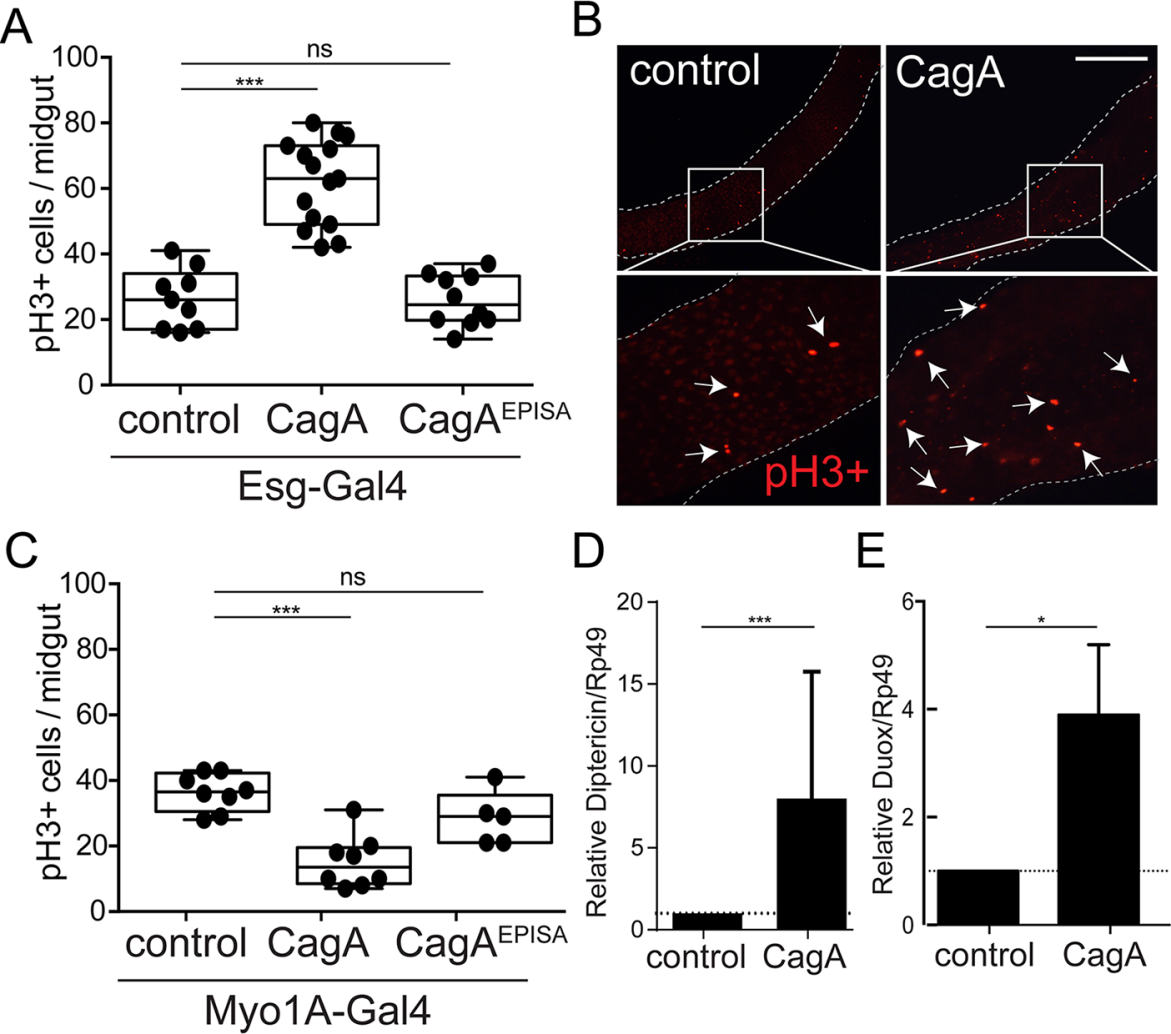
Expression of CagA in adult *Drosophila* intestinal stem cells (ISCs) promotes proliferation and modulates innate immune components. (A, B and C) Cell proliferation shown by anti-phospho histone H3+ cells per adult midgut in flies expressing *UAS-CagA* or *UAS-CagA*^*EPISA*^ in (A) intestinal stem cells (ISCs) using *esg-Gal4*, *UAS-GFP* or (C) enterocytes (ECs) using *Myo1A-Gal4*. (B) Representative image from control and ISC expression of CagA (A), anti-phospho histone H3+ shown in red. Scale bar 200 μm. (C and D) qPCR for expression of *diptericin* (C) and *duox* (D) in the midgut epithelium of control (*esg-Gal4*, *UAS-GFP)* and CagA transgenic *Drosophila*. *P<0.05, ***P<0.0001 and ns, not significant; for all panels, as assessed by a Student’s t-test. For A and C a representative experiment is shown, each point represents one midgut. n>9 flies/genotype per condition. D and E error bar shows the max expression assayed.

CagA has been shown in multiple studies to promote epithelial cell proliferation, and its activity seems to be especially potent in cells with more stem-like characteristics [20]. Recent characterization of *H*. *pylori* micro-colonies in human gastric glands found *H*. *pylori* closely associated with proliferating progenitor cells [21]. Furthermore, experimental infection of mice revealed *H*. *pylori* dependent expansion of Lgr5+ gastric stem cells, with greater expansion occurring with CagA positive *H*. *pylori* [21], suggesting that translocation of CagA into stem cells stimulates their proliferation. Using a transgenic model of CagA expression within the adult *Drosophila* midgut epithelium we examined how expression of CagA within either the intestinal stem cells (ISCs) and their immediate progenitor enteroblasts (EBs) or in the more numerous nutrient absorbing enterocytes (ECs) affects cell proliferation phenotypes. We drove expression of a *UAS-CagA* transgene [18] along with the *UAS-GFP* reporter in the ISC and EB population of the adult midgut using the *escargot-Gal4* driver. We found expression of CagA in this population resulted in high rates of cell proliferation compared to the control (P<0.0001) (Fig 1A and 1B). The observed proliferation in stem cell populations appeared to be dependent upon phosphorylation of CagA, as a non-phosphorylatable version of CagA (CagA^EPISA^) [18] showed proliferation rates similar to that of controls (Fig 1A). In contrast to the effect of CagA expression in stem cells, transgenic expression of CagA or CagA^EPISA^ in enterocytes, using the enterocyte specific driver Myo1A-Gal4, did not induce elevated proliferation in the midgut epithelium and instead was significantly lower than controls (P<0.0001) (Fig 1C). These data show expression of CagA within stem cells, but not enterocytes, of the gut epithelium is sufficient to promote excess cell proliferation in the *Drosophila* midgut.

CagA positive strains of *H*. *pylori* have been shown previously to be sufficient to alter host immune signaling pathways in human patients and animal models [12]. To determine whether expression of CagA in the *Drosophila* midgut is sufficient to alter host immune signaling pathways, we assayed activation of the *Drosophila* intestinal NF-kB pathway, the immune deficiency (IMD) pathway, by measuring expression of pathway-specific target genes encoding antimicrobial peptides, Diptericin, Attacin and Defensin [22,23]. We found the antimicrobial peptide, Diptericin, is overexpressed an average of 8-fold in CagA transgenic flies compared to the control (P<0.0001) (Fig 1D). This was not the case when we expressed the CagA^EPISA^ transgene suggesting immune activation is dependent upon phosphorylation of CagA (S1 Fig). We found both Attacin and Defensin were similarly expressed in controls and CagA or CagA^EPISA^ transgenic flies (S1 Fig), suggesting the overexpression of Diptericin is a specific response to expression of CagA and not the result of non-specific immune activation.

Reactive oxygen species (ROS) have been shown to play an important role in carcinogenesis by inducing DNA damage and promoting proliferation [23,24]. Additionally, CagA-positive strains of *H*. *pylori* have been shown to elicit production of ROS [25]. Therefore, we suspected that similar to its up-regulation of Diptericin, CagA may also activate expression of *Drosophila duox*, a dual oxidase enzyme responsible for translocation of ROS into the midgut lumen [26]. To test this possibility we assayed expression of *duox* by qPCR and found nearly 4-fold higher expression of *duox* in CagA transgenic flies compared to the control (P<0.05) (Fig 1E). Taken together, we conclude that transgenic expression of CagA in the stem cell population of the adult *Drosophila* midgut is sufficient to specifically activate immune signaling pathways previously shown to be associated with *H*. *pylori* infection [27].

Because over-activation of immune signaling pathways is known to be detrimental to long-term survival, we assayed survival of transgenic *Drosophila* to determine if expression of CagA within ISCs affected life span. We found CagA transgenic flies survive similarly to control flies (S1 Fig), indicating expression of CagA within the midgut stem cells has no net negative effect on whole animal survival. Interestingly, flies expressing the CagA^EPISA^ transgene in two independent lines survived significantly longer than CagA-expressing or control flies. This may indicate a previously unappreciated phosphorylation-independent function of CagA.

### CagA transgenic *Drosophila* have an altered gut microbiota that promotes spontaneous cell proliferation within the gut epithelium

**Fig 2:**
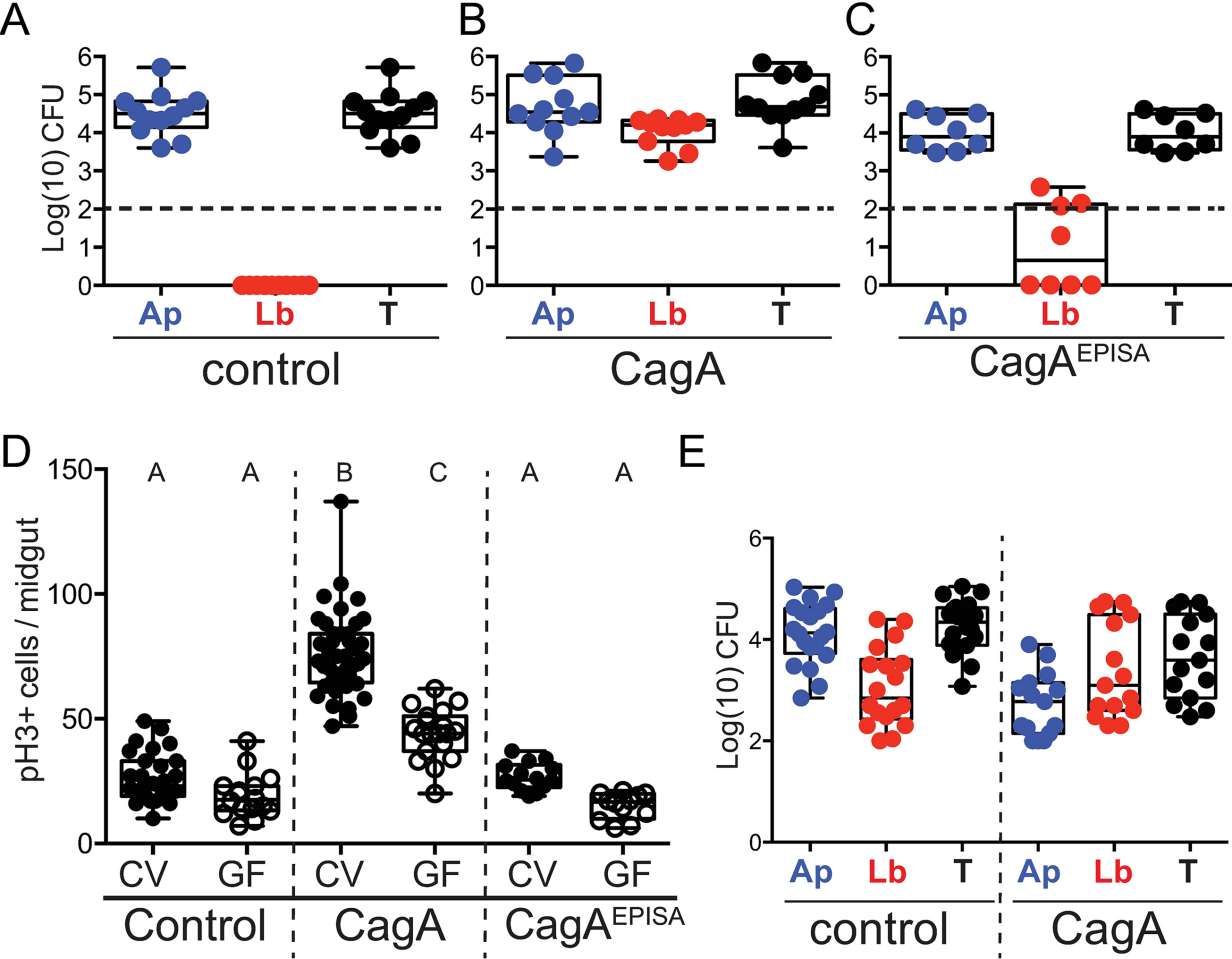
Gut microbiota of CagA transgenic flies is dysbiotic and contributes to cell proliferation in the midgut epithelium. (A-E) Control *(esg-Gal4*, *UAS-GFP)*, CagA (*esg-Gal4*, *UAS-GFP; UAS-CagA*), EPISA (*esg-Gal4*, *UAS-GFP; UAS-CagA*^*EPISA*^). (A-C) Microbial abundance of *Acetobacter pasteurianus* (*Ap*) in blue, *Lactobacillus brevis* (*Lb*) in red, or total (T) in black. CFUs/midgut represented as Log(10) from Control (A), CagA (B) or CagA^EPISA^ (C) adult *Drosophila*. Dashed horizontal line indicates the Limit Of Quantification (LOQ). (D) Cell proliferation shown by anti-phospho histone H3+ cells per midgut in *Drosophila* reared conventionally (CV), closed circles or germ-free (GF), open circles. Conditions that share a letter are not statistically different from each other, as assessed by ANOVA. (E) Microbial abundance of Ap21 and Lb22 from *Drosophila* derived GF then associated with a 3:1 ratio of Ap21:Lb22, shows colonization of *Lb* in control and demonstrates enrichment for *Lb* in CagA transgenic flies.

Dysbiosis of GI microbiota is well recognized as a contributor to both cell proliferation and inflammatory processes known to facilitate the development and progression of intestinal and colonic cancers [4,5]. The gastric microbiota of individuals with *H*. *pylori* infection and gastric cancer is altered from that of control patients [10]. However, whether dysbiosis of gastric microbiota is capable of inducing or contributing to proliferation and disease progression in patients infected with *H*. *pylori* remains uncertain. Additionally, it is unclear whether CagA alone is sufficient to alter microbiota or whether this process would also require *H*. *pylori* itself. To address this question we took advantage of our transgenic model of CagA expression, where no *H*. *pylori* is present, and assayed microbiota of CagA-expressing and control adult *Drosophila*. We dissected the adult midgut of 7-day-old *Drosophila* and plated the contents on MRS agar, a modified nutrient agar commonly used for growth of *Drosophila* gut isolates. Plating revealed that microbiota of the control flies was completely dominated by a single bacterial isolate, whereas microbiota of CagA transgenic flies was made up of this same colony type and another distinct colony morphology. We isolated individual colonies and determined the sequences of their 16S rRNA genes. This analysis identified the single bacterial isolate from control flies as *Acetobacter pasteurianus* (*Ap*) of the *Acetobacteraceae* family (Fig 2A). CagA transgenic flies also contained *Ap* and a second distinct colony type identified as *Lactobacillus brevis* (*Lb*) of the family *Lactobacillaceae* (Fig 2B). Both of these species of bacteria have been previously described as common inhabitants of *Drosophila* intestines in both lab-reared and wild populations [28]. Based on the observed differences in microbiota composition in CagA transgenic flies, we conclude that CagA flies harbor an altered gut microbiota from that of control flies. Additionally, flies expressing the non-phosphorylatable CagA^EPISA^ contained communities dominated by *Ap* (Fig 2C), however, 50% of the flies assayed contained some *Lb* but at much lower levels than were ever observed in CagA transgenic flies. Occasionally we detected additional taxa identified as *L*. *plantarum* and *A*. *tropicalis* in both control and CagA flies, however the presence of those microbes was inconsistent between experiments and also found at variable rates even within flies from the same bottle. Due to their low abundance and inconsistency, we focused on the presence and abundance of *Ap* and *Lb* for our subsequent analysis. Representative strains *A*. *pasteurianus* DORAp21 (Ap21) and *L*. *brevis* DORLb22 (Lb22) were collected from CagA transgenic flies reared under standard conditions and used in subsequent experiments.

The *Drosophila* midgut microbiota is necessary for development and normal homeostatic cell proliferation [29,30], similar to vertebrate gut microbiota [1,31,32]. We therefore asked whether the altered microbiota of CagA transgenic flies could contribute to the excessive cell proliferation observed in these animals by rearing them under germ-free (GF) conditions and assaying cell proliferation. As has been reported previously [33], we found cell proliferation to be slightly higher in conventional (CV) control flies compared to those reared GF (Fig 2D). In the CagA transgenic flies reared GF, we observed a level of cell proliferation intermediate between CV CagA flies and GF control flies (Fig 2D). This suggests that microbiota of CagA transgenic flies contributes to a portion of the total increased cell proliferation observed in CV CagA flies, as compared to CV controls whereas the remaining portion of proliferation is directly due to expression of CagA. We also found that phosphorylation of CagA is required for this microbiota-dependent portion of proliferation, as CagA^EPISA^ flies reared GF showed similar reduction in proliferation to that of GF controls (Fig 2D). These data suggest that the proliferation of midgut cells observed in CagA transgenic flies is multifactorial: expression of CagA induces cell autonomous proliferation and the altered microbiota promotes cell non-autonomous proliferation.

Next we wanted to determine if expression of CagA within the midgut epithelium was responsible for shaping the altered community observed in CagA transgenic flies. *Drosophila* microbiota can be variable and significantly affected by environmental exposure and access to microbial isolates [34]. We aimed to determine whether, given the same inoculum of bacteria, the CagA expressing intestines would assemble a different community from the control intestines. To test this, we derived flies GF and then immediately exposed them to food inoculated with a 3:1 ratio of Ap21:Lb22. This ratio was determined based on our initial analysis of the community structure observed in CagA transgenic flies. Flies were raised to adulthood and then aged seven days to match the experimental setup used in other assays described above. The midgut was dissected and plated on MRS agar and total Colony Forming Units (CFUs) of each bacterial isolate were determined. We found the associated microbiota from control and CagA transgenic flies contained both *Ap* and *Lb* (Fig 2E), confirming that *Lb* can colonize control flies. However, we found the associated microbiota of CagA transgenic flies contained predominantly *Lb* compared to CFU counts for *Ap* (Fig 2D), which deviated significantly from the initial 3:1 (Ap:Lb) inoculum. In addition, the opposite community distribution was seen in control flies, where *Ap* was predominant and the microbial composition more closely matched the CV condition. Taken together these data demonstrate that *Lb* can colonize control flies but that expression of CagA enriches for *Lb*, which could contribute to cell proliferation.

### Activation of innate immune components is a consequence of dysbiotic gut microbiota in CagA transgenic flies

**Fig 3:**
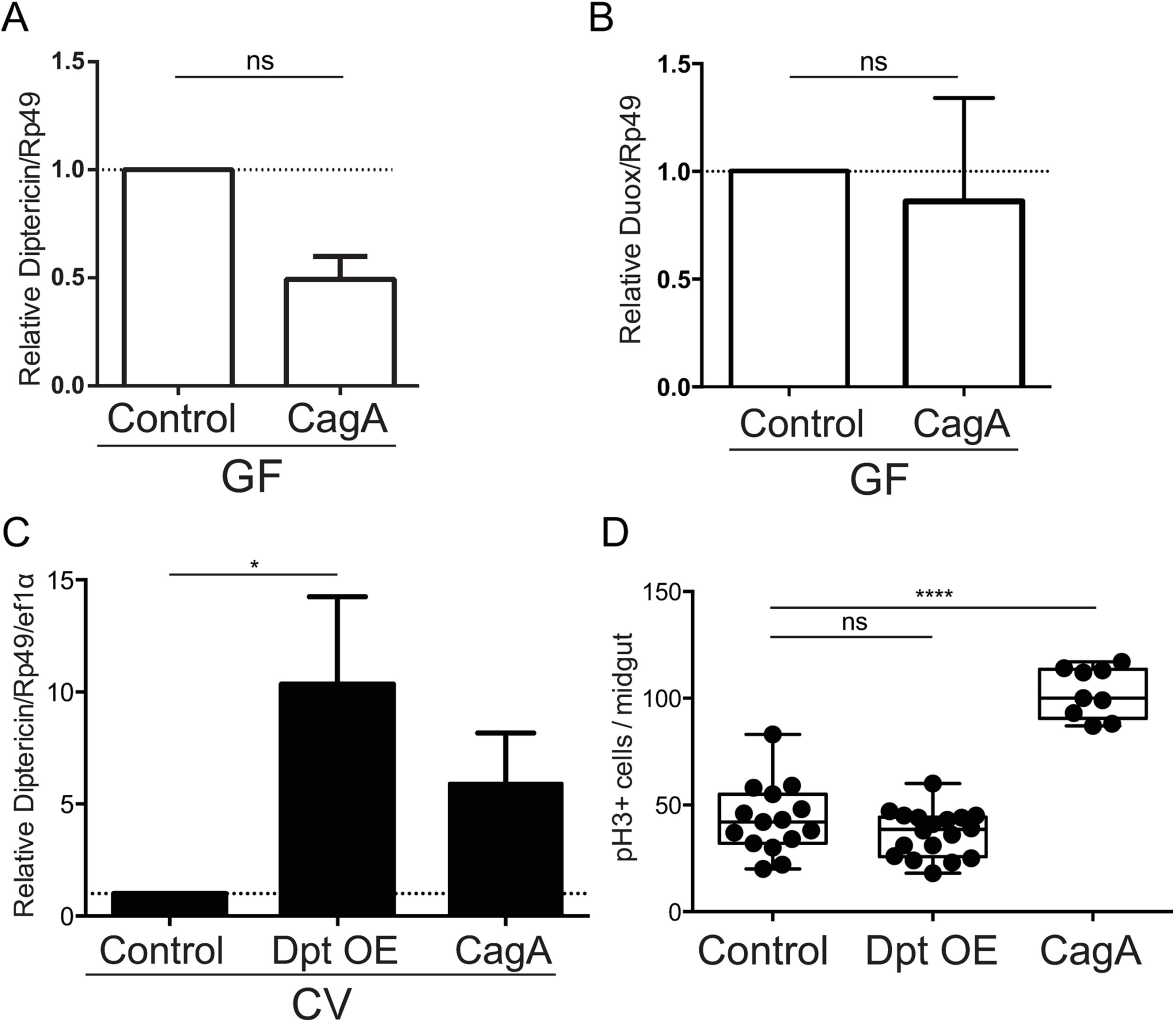
Overexpression of diptericin is not sufficient for proliferation and occurs in response to dysbiotic microbiota in CagA transgenic *Drosophila*. (A and B) qPCR for expression of *diptericin* (A and C) and *duox* (B) control (*esg-Gal4*, *UAS-GFP)* and CagA (*esg-Gal4*, *UAS-GFP*/*UAS-CagA*) transgenic *Drosophila* reared GF. (C and D) Control (as above), Dpt OE (*esg-Gal4*, *UAS-GFP/UAS-Dpt)*, and CagA (as above). qPCR for *diptericin* expression shows overexpression of diptericin similar to levels observed in CagA transgenic flies (C). Cell proliferation shown by anti-phospho histone H3+ cells per adult midgut shows overexpression of Diptericin is not sufficient for cell proliferation in the midgut (D). *P<0.05, **P<0.01 ****P<0.0001 and ns, not significant, as assessed by a Student’s t-test; for all panels.

Flies deficient for IMD pathway activation are more sensitive to infection with pathogenic bacteria and experience overgrowth of commensal communities in the midgut [35,36]. Antimicrobial peptide expression in the midgut is normally reserved for combating pathogenic infection and is not typically induced by commensal microbes [37,38]. Therefore, we reasoned that expression of CagA could alter host immune signaling causing the overexpression of Diptericin and Duox, either of which could be sufficient to alter the microbial community. To test this possibility we reared control and CagA transgenic flies GF and used qPCR analysis to measure expression of Diptericin and Duox in the midgut epithelium. Surprisingly, we found the antimicrobial peptide Diptericin was down-regulated (P<0.01) (Fig 3A), and the ROS transporter Duox (Fig 3B) was not over-expressed in control and CagA transgenic flies in the absence of gut microbiota, suggesting that the observed overexpression of these genes in the CV CagA-expressing flies is a consequence of the dysbiotic microbiota. Because overexpression of Diptericin appears to be dependent on a dysbiotic microbiota, we wanted to determine whether overexpressing Diptericin could explain the microbiota-dependent portion of cell proliferation in CagA transgenic flies. To test this hypothesis we expressed a *UAS*-*diptericin* transgene using *esg-Gal4*, *UAS-GFP*, and then assayed cell proliferation in the midgut epithelium. Using qPCR analysis we first determined that transgenic expression of *diptericin* resulted in similar transcript levels (11-fold over control) to those observed in CV CagA transgenic flies (8-fold over control) (P<0.05) (Fig 3C). When we assayed cell proliferation, we found that flies overexpressing Diptericin in the midgut epithelium showed rates similar to controls and much lower than those observed in CV CagA transgenic flies (P<0.0001) (Fig 3D). Additionally, gut microbiota was not affected in flies overexpressing Diptericin and remained similar to controls (S2 Fig). Taken together we conclude that overexpression of the antimicrobial peptide Diptericin is a consequence rather than a cause of altered gut microbiota and does not contribute to either the increased cell proliferation or the altered microbiota observed in CagA transgenic flies. Because overexpression of the Duox gene is not sufficient for Duox activation at the membrane, we were unable to use similar experiments to test its role in epithelial cell proliferation or dysbiosis.

### Dysbiotic microbiota of CagA transgenic flies is transmissible and sufficient for cell proliferation

**Fig 4:**
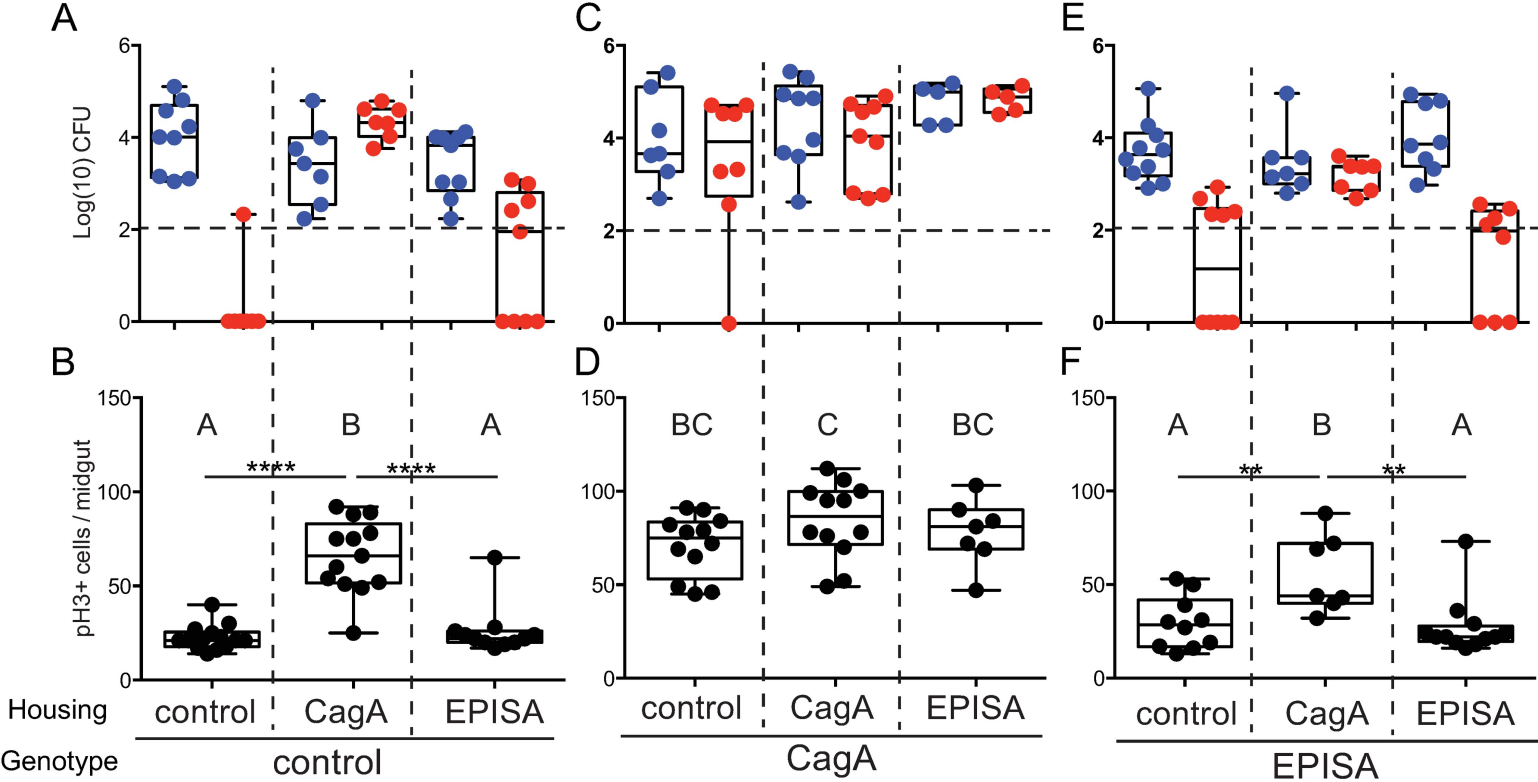
The dysbiotic microbiota of CagA transgenic *Drosophila* is transmissible and promotes pathological proliferation in cohoused *Drosophila*. (A and B) Control *(esg-Gal4*, *UAS-GFP)*, (C and D) CagA (*esg-gal4*, *UAS-GFP; UAS-CagA*) and (E and F) CagA^EPISA^ (*esg-Gal4*, *UAS-GFP; UAS-CagA*^*EPISA*^). (A-F) Newly eclosed conventionally reared adult females were cohoused with age matched control or CagA or CagA^EPISA^ females for seven days. (A, C and E) Microbial abundance of *Acetobacter pasteurianus* (*Ap*) and *Lactobacillus brevis* (*Lb*) observed from dissected adult midgut. Dashed horizontal line indicates the LOQ. (B, D and F) Cell proliferation as shown by anti-phospho histone H3+ cells per midgut. Genotype indicates the genotype assayed, housing indicates the genotype of flies in the cohoused environment.). **P<0.01 ****P<0.0001, as assessed by a Student’s t-test. Groups were also assessed by ANOVA and shared letters indicate groups that are not statistically different from each other.

Based on our findings that the altered gut microbiota of CagA transgenic flies promotes epithelial cell proliferation in the midgut, we predicted that microbiota of CagA transgenic flies would be sufficient to elicit a similar response in control flies. Previous reports in *Drosophila* have shown that the composition of microbial communities in the gut and immediate environment are affected by selective processes within the fly gut [39], therefore we expected cohousing flies might result in transmission of the dysbiotic microbiota and could initiate excessive cell proliferation in control flies. To test this possibility, we reared CagA-expressing and control flies to adulthood under CV conditions and then cohoused them for seven days before we assayed cell proliferation and microbiota community structure. Each condition contained 10–12 females and 8–10 males of a given genotype, and each genotype was cohoused with equivalent numbers of control and CagA or CagA^EPISA^ flies to insure population density was sufficient to support bacterial growth and colonization [39]. To determine whether microbiota of CagA transgenic flies was capable of invading a control microbiota, we first assayed the output microbial community from flies in each housing situation. We found microbiota of control flies cohoused with other controls contained primarily *Ap* and rarely *Lb* (Fig 4A). These flies also showed low rates of cell proliferation (Fig 4B), similar to those observed previously in controls (Fig 1A). However, when control flies were cohoused with CagA flies, we observed both *Ap* and *Lb* in their microbial community (Fig 4A) and we observed a concomitant increase in cell proliferation in the midgut (P<0.0001) (Fig 4B), which was significantly higher than is typically observed in controls. Similarly, CagA^EPISA^ flies cohoused with CagA flies also adopted a CagA-like microbiota of *Ap* and *Lb* (Fig 4E) and we observed higher rates of cell proliferation within the midgut epithelium than when they were housed with control or other CagA^EPISA^ flies (P<0.01) (Fig 4F). We did see sporadically the presence of *Lb* in CagA^EPISA^ flies but the total CFUs were often very low and did not appear to influence epithelial cell proliferation greatly (Fig 4E). Interestingly, cohousing appeared to have no effect on the microbial community of CagA transgenic flies, nor do we see any effect on cell proliferation (Fig 4C and 4D). From these data we conclude that the altered microbiota of CagA transgenic flies is transmissible with the ability to displace a healthy conventional community and promote increased intestinal epithelial cell proliferation even in control flies.

### The pro-proliferative effect of a dysbiotic gut microbiota requires interspecies interactions

**Fig 5:**
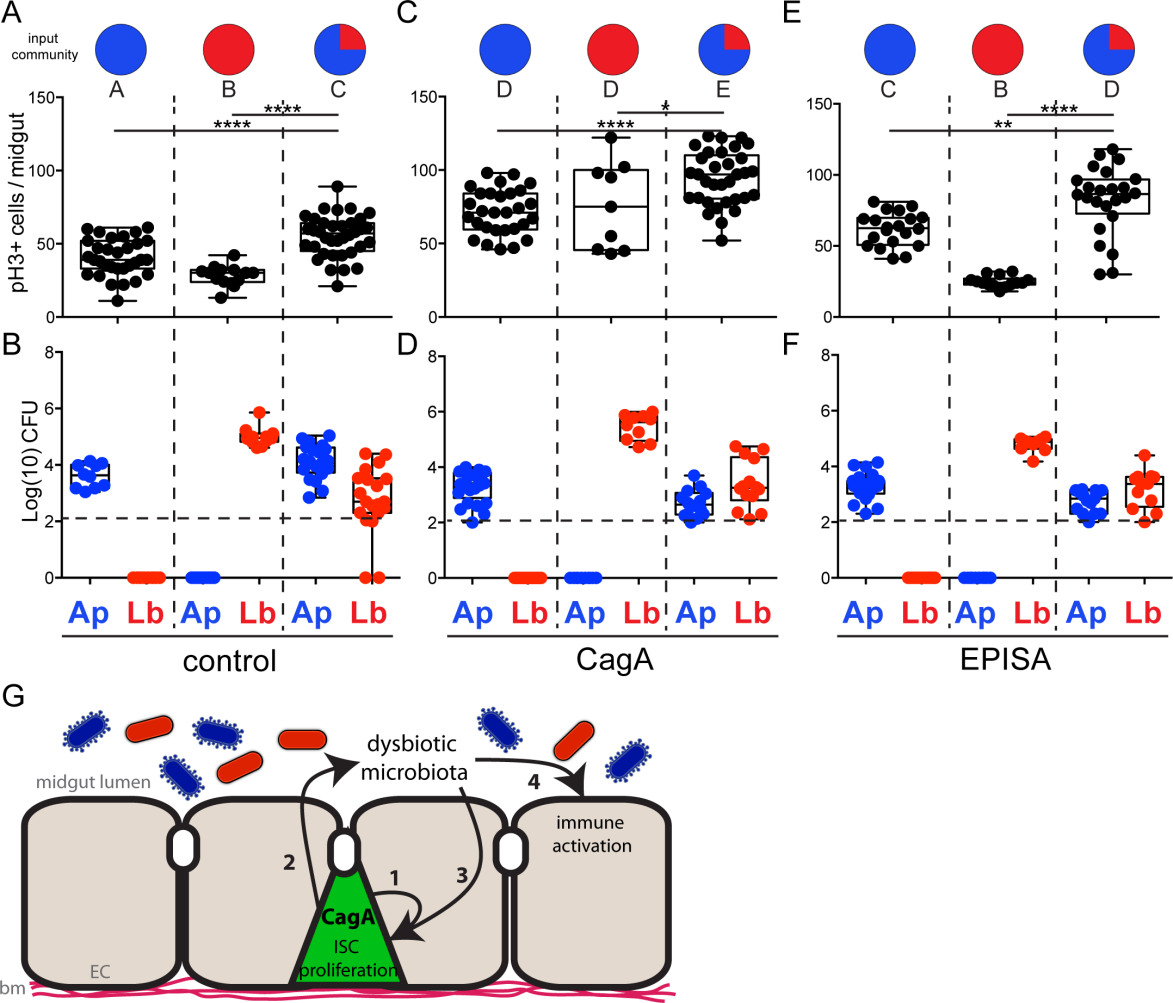
The dysbiotic microbiota of CagA transgenic *Drosophila* requires interspecies interactions to promote proliferation in the midgut epithelium. (A-F) Newly eclosed *Drosophila* reared GF were either mono-associated with *Acetobacter pasteurianus* (Ap21) or *Lactobacillus brevis* (Lb22) or community-associated with both *Acetobacter pasteurianus* (Ap21) shown in red, and *Lactobacillus brevis* (Lb22) shown in blue, indicated as the input community. (A, C and E) Cell proliferation as shown by anti-phospho histone H3+ cells per midgut. (B, D and F) Microbial abundance of *Acetobacter pasteurianus* (Ap21) and *Lactobacillus brevis* (Lb22) observed in the output community seven days post-inoculation. Dashed horizontal line indicates the LOQ. (A and B) Control *(esg-Gal4*, *UAS-GFP)*, (C and D) CagA (*esg-gal4*, *UAS-GFP; UAS-CagA*) and (E and F) CagA^EPISA^ (*esg-Gal4*, *UAS-GFP; UAS-CagA*^*EPISA*^). *P<0.05 **P<0.01 ***P<0.001 ****P<0.0001, as assessed by a Student’s t-test. Groups were also assessed by ANOVA and shared letters indicate groups that are not statistically different from each other. (G) Model of CagA mediated phenotypes in the adult *Drosophila* midgut. Expression of CagA in the intestinal stem cells (ISCs) of the adult midgut promotes cell-autonomous proliferation in the midgut epithelium (1), promotes dysbiosis of midgut microbiota (2), and promotes proliferation (3) and overexpression of innate immune factors (4) in a cell non-autonomous manner through dysbiotic microbiota. Enterocytes (EC), basement membrane (bm).

Next we aimed to identify the specific pro-proliferative component of the dysbiotic microbiota in CagA transgenic flies. *Lactobacillus* was a critical component of the dysbiotic microbiota and is a bacterial genus that has been previously shown to promote intestinal epithelial cell proliferation in *Drosophila* [40,41]. Based on our finding that *Lb* was only present in the CagA pro-proliferative microbiota, we predicted that mono-association with *Lb* might be sufficient for the pathological proliferation observed in CagA transgenic flies. To test this hypothesis we derived control flies GF, raised them to adulthood, and inoculated their food with defined bacterial inocula of Ap21 alone, Lb22 alone, or a 3:1 ratio mixture of Ap21:Lb22, strains isolated from our laboratory CagA transgenic flies. Newly eclosed adult flies were aged for seven days on the defined bacterial inocula before we assayed midgut cell proliferation and the microbial community. First, control flies were mono-associated with Ap21 to recapitulate the CV condition. We found rates of cell proliferation similar to that of CV control flies (Fig 5A), although the final CFUs per midgut were slightly lower (Fig 5B). When control flies were mono-associated with Lb22, rates of proliferation were even lower than those observed upon mono-association with Ap21 or in CV controls (P<0.01) (Fig 5A). We verified Lb22 colonization by plating the dissected midguts and found CFUs were higher than those observed upon Ap21 mono-association (Fig 5B). These data suggest neither Ap21 nor Lb22 alone is sufficient to promote pathological proliferation. In contrast, control flies associated with a 3:1 ratio of Ap21:Lb22 had significantly higher rates of proliferation in the midgut epithelium than control flies mono-associated with either of the individual isolates (P<0.0001) (Fig 5A). We also verified the presence of both microbial isolates from the output community and determined that the total CFUs per midgut recapitulated total CFUs observed in CV CagA flies (Fig 5A). Based on these findings we conclude that the pro-proliferative effects of the dysbiotic CagA microbiota require both *Ap* and *Lb*. This was confirmed when we assayed CagA transgenic and CagA^EPISA^ flies, which were either mono-associated with individual isolates or associated with the CagA-like community of Ap21 and Lb22. In each case, mono-association with either Ap21 or Lb22 caused lower rates of cell proliferation (P<0.01 and P<0.0001) than was observed upon community association with Ap21 and Lb22 (Fig 5C and 5E). We also found that the output microbiota of CagA and CagA^EPISA^ flies associated with Ap21 and Lb22 were similar to CV CagA transgenic flies with an *Lb*-dominate community (Fig 5D and 5F). From these data we conclude that this strain of *L*. *brevis*, Lb22, is not sufficient on its own to promote the excess cell proliferation observed in CagA transgenic flies and instead requires interspecies interactions with Ap21 to become pro-proliferative.

Both *L*. *plantarum* and *L*. *brevis* were previously identified as *Drosophila* commensals that promote epithelial cell proliferation in the *Drosophila* midgut. *L*. *plantarum* strains promote cell proliferation through the stimulation of cellular ROS [41,42] and both species have been shown to stimulate ROS production through release of uracil [40]. We aimed to determine whether the interspecies interactions of the dysbiotic CagA community use these known mechanisms to promote proliferation in the *Drosophila* gut. To test this possibility we reared flies GF and mono-associated flies with a *Drosophila*-derived *L*. *plantarum* (Lp) isolate [43] or a 3:1 ratio of Ap21 and Lp. We found flies associated with Lp alone or the Ap21Lp community showed significantly lower average cell proliferation than was observed with the Ap21Lb22 community association; 30 vs 55 cells/midgut in control, 31 vs 94 cells/midgut in CagA transgenic flies and 29 vs 91 cells/midgut in CagA^EPISA^ transgenic flies (S3A Fig). Additionally, we tested the *Drosophila*-derived *L*. *brevis* strain EW previously shown to elicit intestinal epithelial proliferation and activation of Duox through production of uracil [40]. We found neither mono-association with LbEW or association with a 3:1 ratio of Ap21 and LbEW was sufficient to promote excess proliferation; 26 vs 55 cells/midgut in control, 38 vs 94 cells/midgut in CagA transgenic flies and 25 vs 91 cells/midgut in CagA^EPISA^ transgenic flies (S3B Fig). We concluded that the excessive proliferation promoted by the dysbiotic microbiota of CagA transgenic flies is not dependent upon previously known mechanisms and instead requires unknown interspecies interactions to promote proliferation in the *Drosophila* gut.

## Discussion

Our analysis of the *H*. *pylori* protein CagA, expressed transgenically in the *Drosophila* adult midgut, reveals distinct mechanisms by which this bacterial virulence factor causes excessive epithelial cell proliferation (Fig 5G). First, expression of CagA in intestinal stem cells results in a cell autonomous increase in cell proliferation, independent of the presence of a resident microbiota. This finding is consistent with recent observations of CagA-dependent expansion of gastric stem cells in *H*. *pylori* infected murine gastric glands [21,44]. Our gnotobiotic experiments reveal an additional level of excessive cell proliferation that is mediated through the altered microbiota that assembles specifically in the CagA-expressing adult midgut. This non-cell autonomous cell proliferation is recapitulated in wild type animals that receive a dysbiotic CagA-associated microbial community either through cohousing or through gnotobiotic inoculation with a defined bacterial community.

In addition to the excessive cell proliferation in the CagA-expressing guts, we also observed excessive activation of innate immunity as indicated by up-regulation of genes encoding the antimicrobial peptide, Dpt and the dual oxidase, Duox. This activation, however, does not appear to be the cause, but rather a consequence of the CagA-induced dysbiosis, since it does not occur in CagA-expressing animals when they are reared GF. Furthermore, overexpression of Dpt is not sufficient to cause the dysbiosis or excess cell proliferation observed in CagA-expressing guts. We are actively investigating other mechanisms through which CagA expression leads to dysbiosis.

We only observe CagA-induced excessive cell proliferation when we express the protein in intestinal stem cells and their progenitor enteroblasts, but not when we drive expression in nutrient absorbing enterocytes. During *H*. *pylori* infection of human and murine stomachs, the bacterium is found in close association with gastric stems cells within gastric glands [20], and this physical proximity is correlated with a CagA-induced increase in stem cell proliferation [21]. It will be interesting to dissect the specific functions of stem cells, as opposed to differentiated epithelial cells, that render them more susceptible to CagA-induced proliferation. One possibility is that CagA expression in the stem cells may impair the polarity and epithelial integrity of the resulting tissue. CagA has been shown to disrupt epithelial polarity in cultured monolayers [45,46], and we noted numerous genetic interactions between CagA and polarity genes in a screen for modifiers of CagA-induced *Drosophila* retinal epithelial morphology disruption [47].

### Dysbiosis and Helicobacter-associated disease

Gastrointestinal dysbiosis, as analyzed in fecal microbiome samples, is strongly linked to colorectal cancer risk [6], however the effect of dysbiosis within gastric communities is less clear. *H*. *pylori* infection itself can be viewed as a gastric dysbiosis characterized by overgrowth of a pathobiont that is a known carcinogen. *H*. *pylori* infection is also associated with other alterations in the gastric microbial ecosystem. Patients infected with *H*. *pylori* show altered gastric microbiota from that of uninfected individuals [20] and those changes revert back to an uninfected state upon *H*. *pylori* eradication [14]. Similarly, several groups have reported altered gastric microbiota in *H*. *pylori* infected versus uninfected mice [48,49]. Although the mechanism that could induce gastric dysbiosis in *H*. *pylori* infected individuals remains unknown, increased gastric pH, decreased gastric motility and gastric atrophy have all been proposed [50]. Our results are the first to specifically implicate CagA in contributing to microbiota alterations upon *H*. *pylori* infection. We plan to use our model of CagA expression within the *Drosophila* midgut to investigate the role of pH in microbiota maintenance and dysbiosis.

Whether *H*. *pylori*-induced microbiota shifts contribute to this bacterium’s pathogenesis in humans is not known. However, experiments in mice have demonstrated that pre-infection microbiota composition modulates the severity of *H*. *pylori*-induced pathology [15]. Furthermore, experiments in GF and CV gastrin-overexpressing mice demonstrated that the presence of the microbiota accelerated *H*. *pylori*-induced stomach cancer in this model [16], and that specific bacterial taxa from these *H*. *pylori* infected mice were sufficient to induce disease acceleration [51].

### Microbial interspecies interaction mediate host pathology

Dysbiosis is usually associated with either the overgrowth of a single pathobiont, such as adherent-invasive *Escherichia coli* in a TLR5 deficient model of spontaneous colitis [52], or the loss of a single protective strain, such as the anti-inflammatory *Faecalibacterium prausnitzii* that is reduced in Crohn’s disease patients [53]. In contrast, the dysbiotic microbiota of CagA transgenic *Drosophila* requires the presence of two bacteria, *Acetobacter pasteurianus* (*Ap*) and *Lactobacillus brevis* (*Lb*). These species of *Acetobacter* and *Lactobacillus* are well-known members of the *Drosophila* midgut microbiota [28], and interspecies interactions between closely related species have been shown to determine nutrient allocation in the fly [43].

*Lactobacillus* species have been previously shown to promote epithelial proliferation in *Drosophila* via induction of epithelial ROS production [41,42], in some circumstances via bacterial derived uracil [40]. We do not suspect these known mechanisms to be the cause of excess cell proliferation resulting from dysbiotic CagA microbiota because neither association with a ROS-inducing *L*. *plantarum* or a uracil-producing *L*. *brevis* (LbEW) elicited the high levels of proliferation we observed with the Ap21Lb22 community. Our data instead suggest a novel mechanism, which requires interspecies interactions between the specific *Ap* and *Lb* strains we isolated from our *Drosophila* colony. Metabolic cross feeding between *Acetobacter* and *Lactobacillus* species has been well described (e.g. [54]) and it is plausible to imagine that the microbial metabolites produced by these strains during mono-association in the *Drosophila* midgut differ significantly from those produced when both strains are present. We are currently exploring the genomic and metabolic properties of these bacterial strains. To our knowledge, this is the first reported demonstration of an interspecies interaction being the etiological agent of dysbiosis-associated disease.

### Virulence factor induced dysbiosis as a contributor to bacterial pathogenesis

Based on our results, we propose a new paradigm for bacterial pathogenesis by which bacterial virulence factors induce dysbiosis that contributes to disease pathology. This view expands the traditional view of virulence factors as modifiers of host cell biology and considers their capacity to modify host microbial ecology. *Salmonella enterica* serovar Typhimurium provides an exemplar of this mechanism as a pathogen that promotes intestinal inflammation as an adaptive metabolic strategy [3]. A consequence of this host inflammation, which requires the *invA* and *spiB* virulence factors, is the expansion of other pro-inflammatory Proteobacteria that promote disease pathology [55]. An implication of this view of bacterial virulence factors is that treating the pathogen-associated dysbiosis could mitigate the pathology of infectious disease. With a dwindling arsenal of effective antibiotics, future treatments for infectious disease may rely more heavily on therapies such as fecal microbiota transplants and probiotics. Simple model systems for dissecting the mechanisms of dysbiosis will provide useful tools for advancing these therapeutic approaches.

## Material and methods

### Cultivation of *Drosophila* and bacteria

*Drosophila* melanogaster (*Wolbachia-*free) were reared at 25°C, 12h:12h light:dark cycle in a humidified chamber on standard cornmeal agar medium. All assays were performed on mated adult females. The following *Drosophila* lines were used: Sp/CyO; *UAS-CagA*, *UAS-CagA; Dr/TM3 Sb* and *UAS-CagA*^*EPISA*^ [18] *w; UAS-Dipt*,*imd*,*DiptD/CyO; spz/TM3 Sb*, [22], *esg-Gal4*, *UAS-GFP; Dr/TM6B Tb* [56] and *Myo1A-Gal4* [57]. *Drosophila* gut microbiota members were isolated on MRS agar from aseptically dissected 7–10 day old adult female guts.

### Axenic and gnotobiotic fly preparation

Fresh laid eggs (<18 hr old) were collected from apple juice agar plates and dechorionated in 50% bleach for 3 minutes then rinsed in 2 consecutive washes of 70% ethanol followed by 2 washes in sterile H_2_O. Sterile embryos were then aseptically transferred to sterile fly food and maintained at 25°C. Inocula for gnotobiotic flies were prepared as follows and added to the food after aseptic transfer of eggs: 100 μl of cell suspension were added to each gnotobiotic vial to give 5 x 10^6^ cells per vial. For inculcation with the two species community microbes were added in a 3:1 ratio of *Ap*:*Lb* to make up the total inoculum. Strains used: *A*. *pasteurianus* DORAp21, *L*. *brevis* DORLb22, *L*. *brevis* EW [40] and *L*. *plantarum* [43]. Axenia was confirmed by homogenizing a single representative larva from each bottle in 200 μl sterile 1X PBS. Homogenates were plated on MRS agar and incubated at 30°C for 2–4 days to evaluate bacterial growth. Axenic flies were transferred to sterile fly food 0–2 days after eclosing and allowed to age for 7 days at 25°C. As has been previously reported [58,59] axenic flies took longer to eclose than CV flies. We noted a similar delay in eclosure with Lb mono-associated flies. We also noted slight delays in ecolsure of CV CagA transgenic flies, which may reflect the significant Lb fraction of their microbiota. All data shown represent data collected from 7–10 day old mated adult females.

### CFU determination

Microbial density was determined to assess the presence and/or abundance of each bacterial species associated with the host. In all experiments 7–10 day old adult female flies were dissected in sterile 1X PBS. The dissected midgut was immediately placed in 200μl 1X PBS and homogenized with a handheld pestle grinder for 20–30 seconds/gut. The resulting homogenate underwent serial dilutions and was then plated on MRS agar plates using sterile glass beads and incubated at 30°C for 2–4 days under aerobic conditions. CFU counts were determined after manually counting each plate. The limit of quantification (LOQ) was defined as 200 CFU per plate [60].

### Immunostaining

Aseptically-dissected 7–10 day old female guts were dissect in sterile 1X PBS then fixed for 30 minutes to 1 hour in fresh 4% Paraformaldehyde/1X PBS. Guts were washed 3 times for 15 minutes with 1X PBS containing 0.1% Triton X-100 (PBST) then blocked with the same solution plus 0.02% BSA (PBSTB) for 30 minutes at room temperature. Primary antibodies were applied either for 2 hours at room temperature or overnight at 4°C and include Rabbit anti-phospho histone H3 (1:500; Millipore) and Chicken anti-GFP (1:500; AVES labs). Guts were then washed 3 times for 15 minutes with PBSTB and incubated with AlexaFluor 594 Goat anti-Rabbit and AlexaFluor 488 Goat anti-Chicken, for 2 hours at room temperature. Guts were then washed 3 times for 15 minutes with PBSTB and mounted on glass slides with ProLong Diamond with DAPI anti-fade mounting media (Life Technologies). pH3+ cells were counted manually on a Nikon compound microscope. Total cell count includes pH3+ cells from the base of the proventriculus to midgut/hindgut junction at the posterior end of the midgut.

### Identification of bacterial species

Bacteria were grown statically (*Lactobacillus)* or shaking (*Acetobacter)* at 30°C to late-log phase and genomic DNA was isolated using the Qiagen DNeasy Blood and Tissue Kit. Lysozyme digestion was used as a pre-treatment procedure. The University of Oregon Genomics Core Facility performed amplification of the V4 variable region of the 16S Ribosomal gene and sequenced products. The resulting sequences were submitted to a standard nucleotide BLAST that identified isolates as *A*. *pasteurianus*, which we gave the strain name DORAp21 and *L*. *brevis*, which we gave strain name DORLb22.

### Quantitative RT-PCR

Total RNA was extracted from 5 pooled adult female midguts/per sample, using TRIzol reagent and the Qiagen RNeasy Mini Kit according to manufacturer’s protocol. cDNA was prepared using the Thermo Fisher Scientific SuperScript III Reverse Transcriptase Kit. cDNA was analyzed using gene-specific primers in triplicate, for at least three independent experiments. Data were analyzed by relative quantification by normalization to the gene *rp49*. Primers sequences were previously published [37] and are listed below: rp49: Forward 5’AGA TCG TGA AGA AGC GCA CCA AG 3’ Reverse 5’ CAC CAG GAA CTT CTT GAA TCC GG 3’; Diptericin: Forward 5’ GGC TTA TCC GAT GCC CGA CG 3’ Reverse 5’ TCT GTA GGT GTA GGT GCT TCC C 3’; Duox: Forward 5’ GCT GCA CGC CAA CCA CAA GAG ACT 3’ Reverse 5’ CAC GCG CAG CAG GAT GTA AGG TTT-3’; Attacin: Forward 5’ ACG CCC GGA GTG AAG GAT GTT 3’ Reverse 5’ GGG CGA TGA CCA GAG ATT AGC AC 3’; Defensin: Forward 5’ TGC AGC ATA GCC GCC AGA A 3’ Reverse 5’ TTG CAG TAG CCG CCT TTG AAC C3’.

### Adult survival assay

<24 hour-old adult flies were collected and placed on fresh food vials and left to mate for 24 hours. Mated adults were then sorted into 3-vials of 20 females and 20 males each, with 3 replicates of each genotype. Vials were scored 3 times per week and the number of dead flies was recorded each day until all the flies were recorded dead or the vial was empty.

## Supporting information

S1 Dataset Excel spreadsheet containing data values plotted in main and supporting figures. (XLSX)

S1 Fig Expressing CagA in the ISCs of the midgut does not affect expression of antimicrobial peptides Attacin or Defensin nor does it have any effect on whole animal survival. (A-C) Control (*esg-Gal4*, *UAS-GFP)*, CagA (*esg-Gal4*, *UAS-GFP*/*UAS-CagA*) or CagA^EPISA^ (*esg-Gal4*, *UAS-GFP; UAS-CagA*^*EPISA*^) transgenic *Drosophila*. (A and B) q-PCR data showing the anti-microbial peptides, Attacin (A) and Defensin (B) are expressed normally in midgut epithelium of conventionally reared *Drosophila*. (C) Survival curve using Kaplan-Meier estimate of survival in conventionally reared *Drosophila*. Note; *Drosophila* expressing the CagA^EPISA^ transgene survive significantly longer than even control flies suggesting unappreciated cellular interactions may occur with this non-phosphorylatable version of CagA. This phenomenon was observed with two independent lines expressing the CagA^EPISA^ transgene.
(TIF)

S2 Fig Overexpression of antimicrobial peptide diptericin is not sufficient to alter host microbiota. (A) Control (*esg-Gal4*, *UAS-GFP)*, CagA (*esg-Gal4*, *UAS-GFP*/*UAS-CagA*) or Dpt OE (*esg-Gal4*, *UAS-GFP/UAS-dpt*) transgenic *Drosophila*. Microbial abundance assay reveals CFUs/midgut of *Acetobacter pasteurianus* (*Ap*) and *Lactobacillus brevis* (*Lb*) in control, CagA and Dpt OE *Drosophila* reveals overexpression of Diptericin is not sufficient to alter host microbiota.
(TIF)

S3 Fig *Lactobacillus* species *plantarum* and *brevis* EW fail to elicit high rates of cell proliferation observed with CagA microbiota; *Acetobacter pasteurianus* DORAp21 and *Lactobacillus brevis* DORLb22. (A and B) Control *(esg-Gal4*, *UAS-GFP)*, CagA (*esg-Gal4*, *UAS-GFP; UAS-CagA*), CagA^EPISA^ (*esg-Gal4*, *UAS-GFP; UAS-CagA*^*EPISA*^) transgenic *Drosophila*. (A) Flies were mono-associated with *Lactobacillus plantarum* (Lp) or a 3:1 ratio of Ap21:*Lp*. No significant difference between any group was observed. (B) Flies were mono-associated with *Lactobacillus brevis* EW (LbEW) or a 3:1 ratio of Ap21:LbEW. Proliferation was calculated based on incorporation of phospho-histone H3. *p<0.05, **p<0.01 and ns, not significant; for all panels.
(TIF)
