# Supplementary figures and images for: The bacterial virulence factor CagA induces microbial dysbiosis that contributes to excessive epithelial cell proliferation in the Drosophila gut
Source: PLoS Pathog. 2017 Oct 19;13(10):e1006631. doi: 10.1371/journal.ppat.1006631 (PMC5648253; doi:10.1371/journal.ppat.1006631)

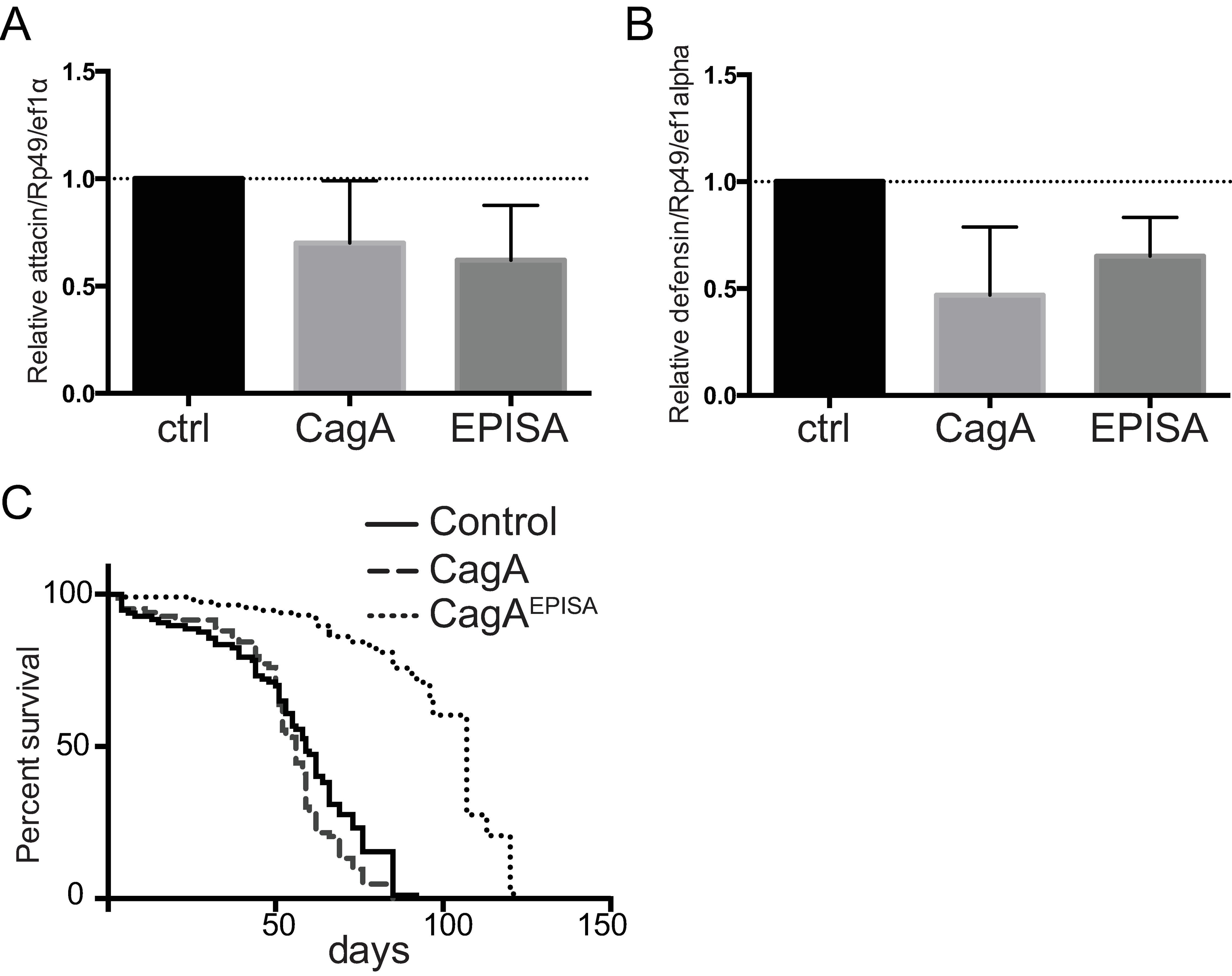

Supplement: S1 Fig — (A-C) Control (esg-Gal4, UAS-GFP), CagA (esg-Gal4, UAS-GFP/UAS-CagA) or CagAEPISA (esg-Gal4, UAS-GFP; UAS-CagAEPISA) transgenic Drosophila. (A and B) q-PCR data showing the anti-microbial peptides, Attacin (A) and Defensin (B) are expressed normally in midgut epithelium of conventionally reared Drosophila. (C) Survival curve using Kaplan-Meier estimate of survival in conventionally reared Drosophila. Note; Drosophila expressing the CagAEPISA transgene survive significantly longer than even control flies suggesting unappreciated cellular interactions may occur with this non-phosphorylatable version of CagA. This phenomenon was observed with two independent lines expressing the CagAEPISA transgene. (TIF) [file ppat.1006631.s002.tif]

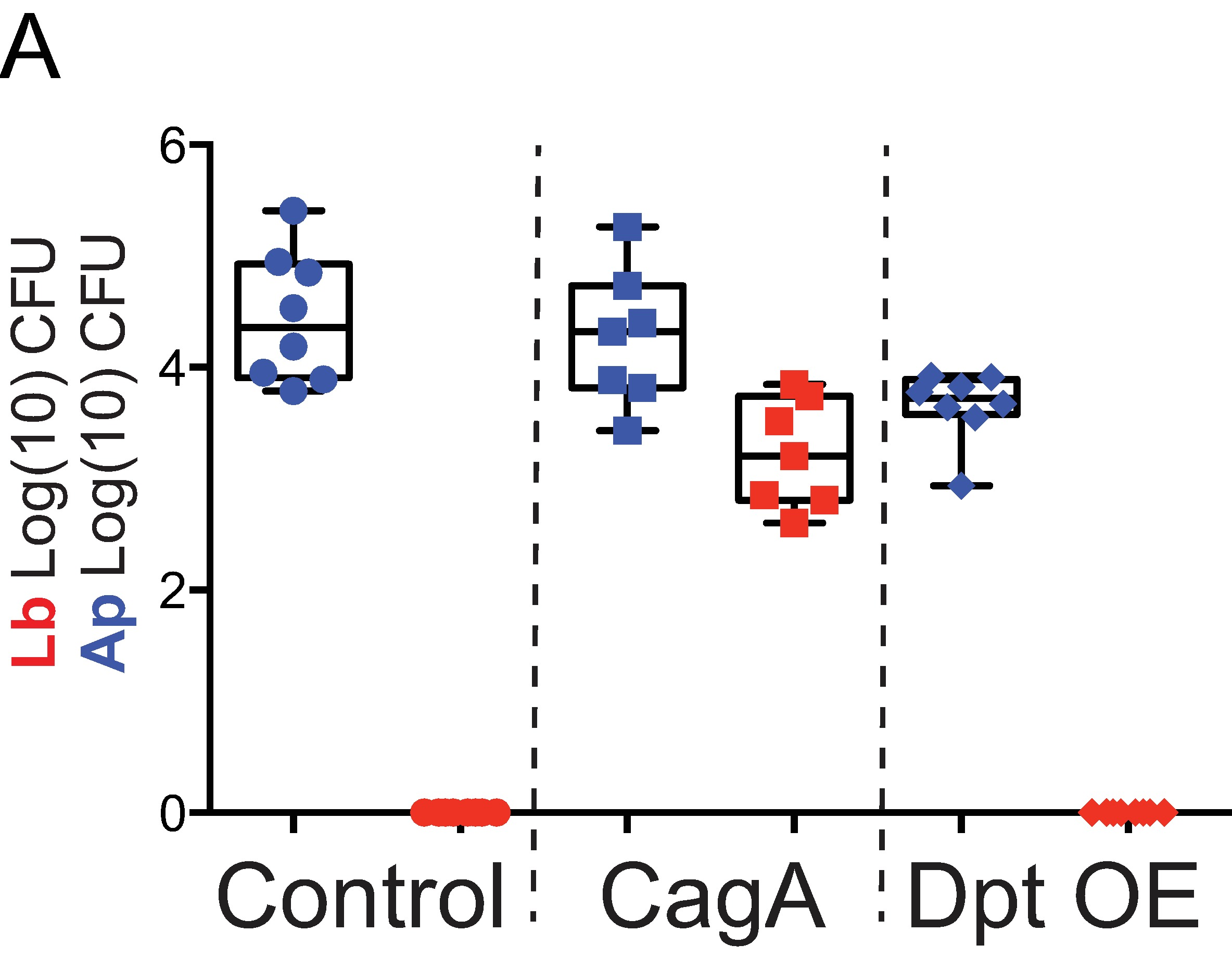

Supplement: S2 Fig — (A) Control (esg-Gal4, UAS-GFP), CagA (esg-Gal4, UAS-GFP/UAS-CagA) or Dpt OE (esg-Gal4, UAS-GFP/UAS-dpt) transgenic Drosophila. Microbial abundance assay reveals CFUs/midgut of Acetobacter pasteurianus (Ap) and Lactobacillus brevis (Lb) in control, CagA and Dpt OE Drosophila reveals overexpression of Diptericin is not sufficient to alter host microbiota. (TIF) [file ppat.1006631.s003.tif]

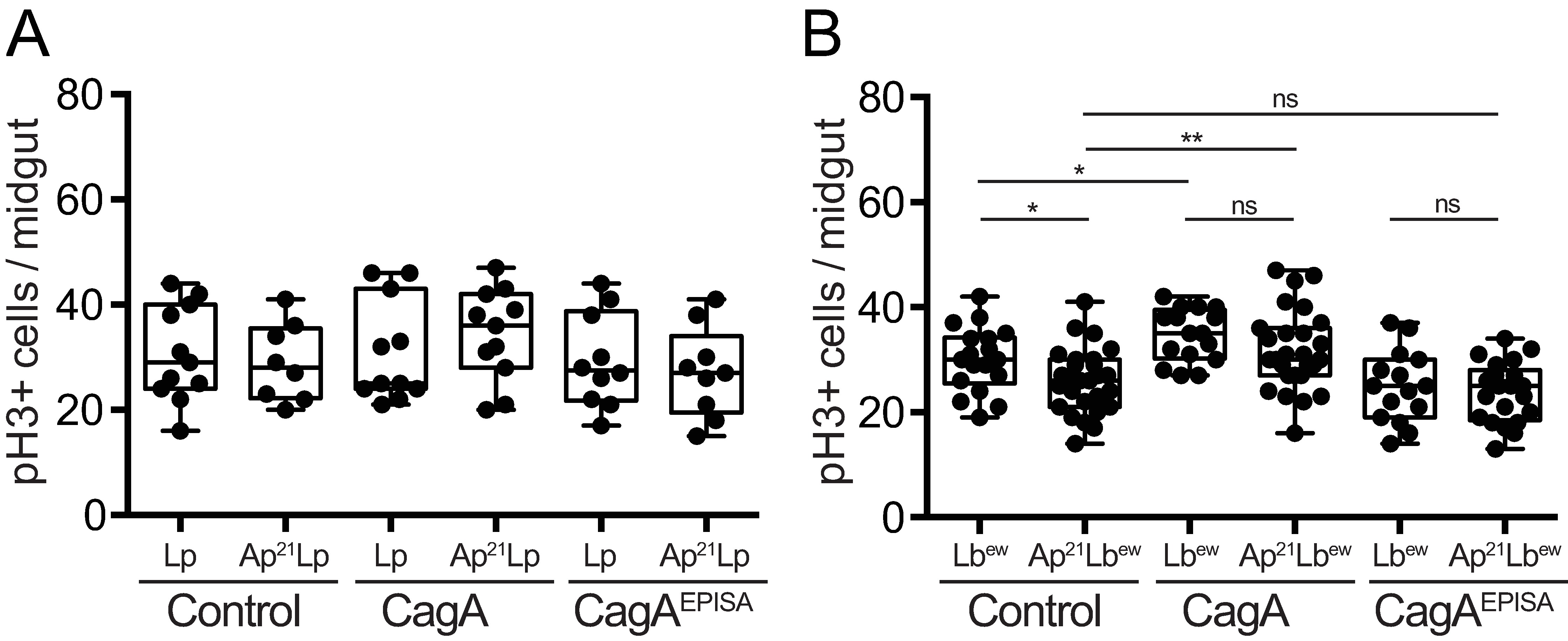

Supplement: S3 Fig — (A and B) Control (esg-Gal4, UAS-GFP), CagA (esg-Gal4, UAS-GFP; UAS-CagA), CagAEPISA (esg-Gal4, UAS-GFP; UAS-CagAEPISA) transgenic Drosophila. (A) Flies were mono-associated with Lactobacillus plantarum (Lp) or a 3:1 ratio of Ap21:Lp. No significant difference between any group was observed. (B) Flies were mono-associated with Lactobacillus brevis EW (LbEW) or a 3:1 ratio of Ap21:LbEW. Proliferation was calculated based on incorporation of phospho-histone H3. *p<0.05, **p<0.01 and ns, not significant; for all panels. (TIF) [file ppat.1006631.s004.tif]
